# Endovascular treatment considerations in Idiopathic Intracranial Hypertension (IIH)

**DOI:** 10.1186/2045-8118-12-S1-O61

**Published:** 2015-09-18

**Authors:** Heinke Pulhorn, Arun Chandran, Mani Puthuran, Hans Nahser, Catherine McMahon

**Affiliations:** 1 The Walton Centre, UK

## Introduction

Impaired cerebral venous sinus outflow leading to cerebral venous hypertension has been implicated as a potential final common pathway in the pathophysiology of idiopathic intracranial hypertension (IIH). The aim of this study is to assess the role of endovascular management strategies in the form of either primary venous sinus angioplasty or venous stenting for refractory IIH.

## Method

Retrospective study of 37 consecutive patients with refractory IIH and imaging evidence of cerebral venous sinus outflow impairment. Primary venous angioplasty or secondary stenting were performed and clinical outcomes were documented on a standardised proforma.

## Results

20 out of the 37 cases showed positive pressure gradients and had endovascular management where there was variable reduction of the pressure gradient. 17 (of whom 12 had only sinus venoplasty and 5 had venoplasty followed by sinus stenting) out of 20 showed clinical improvement or resolution of symptoms. 3 patients were refractory to endovascular management and stabilised after ventriculo-peritoneal shunting.

## Conclusion

The pathophysiology of IIH from venous hypertension secondary to venous outflow impairment is controversial. A selected group of patients with IIH and cerebral venous outflow impairment can benefit from endovascular treatment. In our experience 60% of patients showed clinical improvement with primary sinus venous angioplasty alone. This is a potential alternative to CSF shunting or primary stenting of venous sinus. After additional venous sinus stenting of refractory cases 85% of our patients in our cohort improved clinically.
